# Author Correction: Angiotensin-converting enzyme inhibitor promotes angiogenesis through Sp1/Sp3-mediated inhibition of notch signaling in male mice

**DOI:** 10.1038/s41467-023-38092-6

**Published:** 2023-04-25

**Authors:** Hanlin Lu, Peidong Yuan, Xiaoping Ma, Xiuxin Jiang, Shaozhuang Liu, Chang Ma, Sjaak Philipsen, Qunye Zhang, Jianmin Yang, Feng Xu, Cheng Zhang, Yun Zhang, Wencheng Zhang

**Affiliations:** 1 The Key Laboratory of Cardiovascular Remodeling and Function Research, Chinese Ministry of Education, Chinese National Health Commission and Chinese Academy of Medical Sciences, The State and Shandong Province Joint Key Laboratory of Translational Cardiovascular Medicine, Department of Cardiology, Qilu Hospital of Shandong University, Cheeloo College of Medicine, Jinan, China; 2 Department of Obstetrics and Gynecology, LiaoCheng People’s Hospital, LiaoCheng, China; 3 Department of Bariatric and Metabolic Surgery, General Surgery, Qilu Hospital of Shandong University, Cheeloo College of Medicine, Jinan, China; 4 Department of Cell Biology, Erasmus MC, Rotterdam, The Netherlands; 5 Department of Emergency Medicine, Chest Pain Center, Shandong Provincial Clinical Research Center for Emergency and Critical Care Medicine, Qilu Hospital of Shandong University, Jinan, China

**Keywords:** Angiogenesis, Transcription, Predictive markers

Correction to: *Nature Communications* 10.1038/s41467-023-36409-z, published online 9 February 2023

In this article the grant number ZR2020JQ30 relating to Natural Science Foundation for Distinguished Young Scholars of Shandong Province for Wencheng Zhang was omitted.

In addition, the grant numbers 81970198, 81970366, 82030051 relating to the National Natural Science Foundation of China for Wencheng Zhang, Jianmin Yang and Yun Zhang respectively were omitted.

The grant number 2021YFA1301102 relating to National Key Research and Development Project of China for Qunye Zhang was also omitted.

The original article has been corrected.

