# Supplementary material for: Angiotensin-converting enzyme inhibitor promotes angiogenesis through Sp1/Sp3-mediated inhibition of notch signaling in male mice
Source: Nat Commun. 2023 Feb 9;14:731. doi: 10.1038/s41467-023-36409-z (PMC9911748; doi:10.1038/s41467-023-36409-z)
Supplement: Supplementary file 1 — Supplementary Information [file 41467_2023_36409_MOESM1_ESM.pdf]

Table S1. Primer sequences used in this study.

Gene name	Primer sequences for qPCR (5'-3')
Human Notch1	Forward: GAGGCGTGGCAGACTATGC
	Reverse: CTTGTACTCCGTCAGCGTGA
Human HES1	Forward: AGCACACTTGGGTCTGTGC
	Reverse: TGAAGAAAGATAGCTCGCGG
Human HEY1	Forward: ATCTGCTAAGCTAGAAAAAGCCG
	Reverse: GTGCGCGTCAAAGTAACCT
Human DLL4	Forward: CTGGAGCTCAGCGAGTGTGAC
	Reverse: CCTGGTCCTTACAGCTGCCTC
Human β-actin	Forward: GGAAATCGTGCGTGACATTAA
	Reverse: AGGAAGGAAGGCTGGAAGAG
Human Notch1 (ChIP)	Forward: TACTGGTGATTCTCCTGGCA
	Reverse: CAGGGCCGCGCCGGGGGCGG
Mouse Notch1	Forward: GATGGCCTCAATGGGTACAAG
	Reverse: TCGTTGTTGTTGATGTCACAGT
Mouse HES1	Forward: CCAGCCAGTGTCAACACGA
	Reverse: AATGCCGGGAGCTATCTTTCT
Mouse HEY1	Forward: GCGCGGACGAGAATGGAAA
	Reverse: TCAGGTGATCCACAGTCATCTG
Mouse DLL4	Forward: TTCCAGGCAACCTTCTCCGA
	Reverse: ACTGCCGCTATTCTTGTCCC
Mouse VE-cadherin	Forward: CACTGCTTTGGGAGCCTTC

	Reverse: GGGGCAGCGATTCATTTTTCT
Mouse VE-cadherin	Forward: CACTGCTTTGGGAGCCTTC
	Reverse: GGGGCAGCGATTCATTTTTCT

Supplemental Figures

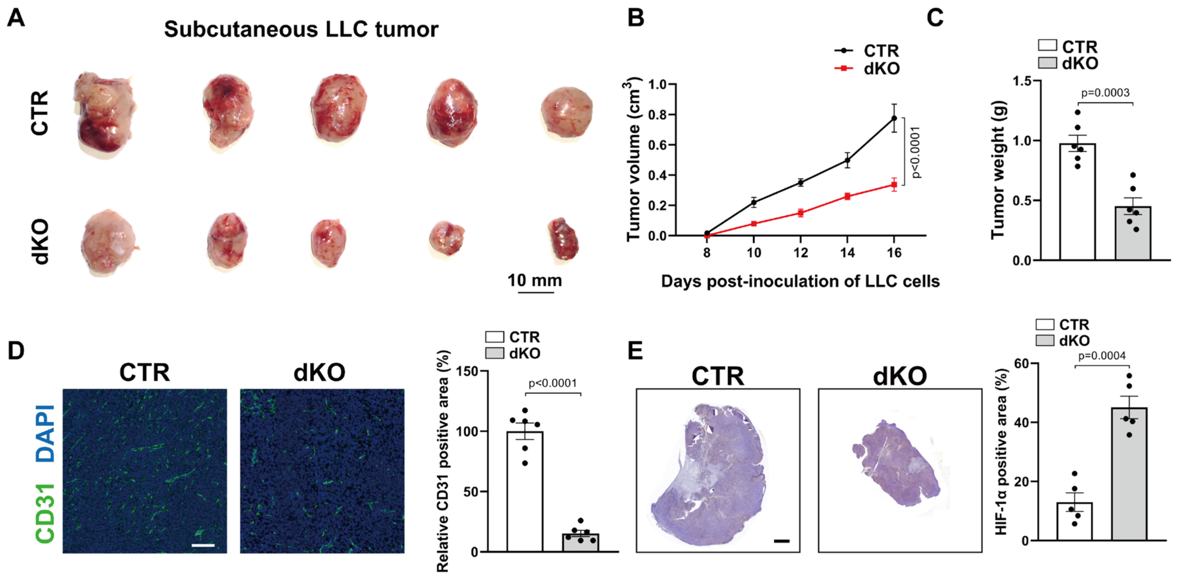

Fig. S1. Endothelial Sp1/Sp3 is necessary for LLC tumor growth and angiogenesis.

A, Representative images of subcutaneous LLC tumors in CTR and dKO mice. Scale bar: 10 mm. **B**, The volume of subcutaneous LLC tumors from CTR and dKO mice measured on days 8, 10, 12, 14, and 16. n = 6. **C**, The weight of subcutaneous LLC tumors from CTR and dKO mice measured on day 16. n = 6. **D**, Representative images of immunofluorescence staining of $CD31^+$ capillaries and quantification of $CD31^+$ areas in subcutaneous LLC tumors from CTR and dKO mice on day 16. Scale bar: 100 μm. n = 6. **E**, Representative images of immunohistochemistry (IHC) staining of HIF-1α and quantification of HIF-1α^+ area in subcutaneous LLC tumors from CTR and dKO mice on day 16. Scale bar: 1 mm. n = 5. Two-way ANOVA followed by Bonferroni multiple-comparison analysis for **B**. Two-tailed Student's unpaired t-test for **C, D** and **E**. Data are presented as mean ± SEM. Source data are provided as a Source Data file.

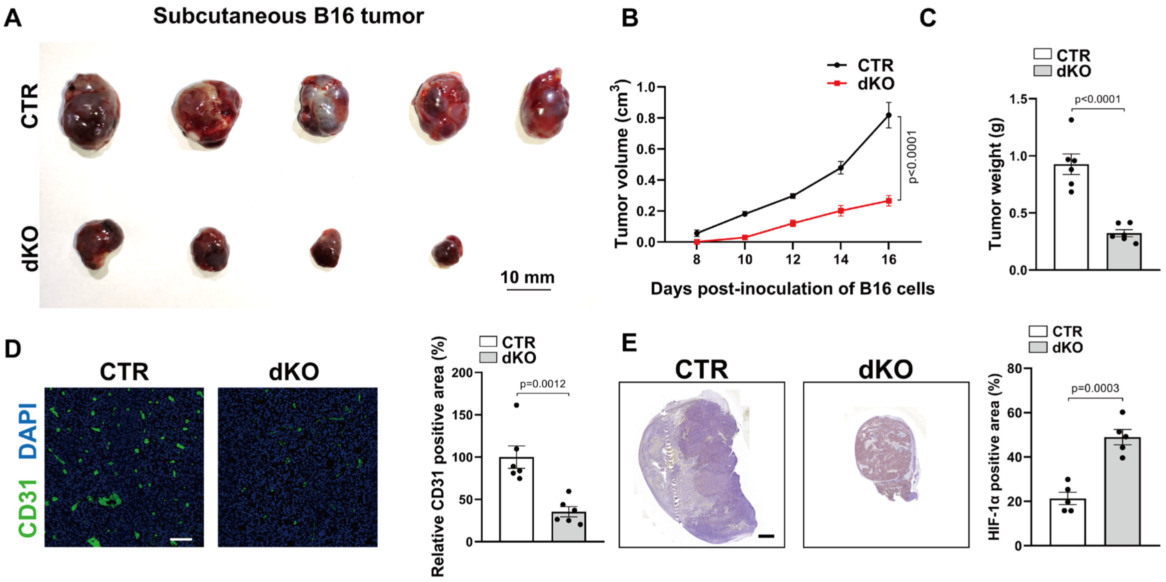

Fig. S2. Endothelial Sp1/Sp3 is essential for B16 tumor growth and angiogenesis.

A, Representative images of subcutaneous B16 tumors in CTR and dKO mice. Scale bar: 10 mm. **B**, The volume of subcutaneous B16 tumors from CTR and dKO mice on days 8, 10, 12, 14, and 16. n = 6. **C**, The weight of subcutaneous B16 tumors from CTR and dKO mice on day 16. n = 6. **D**, Representative images of immunofluorescence staining of CD31$^+$ capillaries and quantification of CD31$^+$ areas in subcutaneous B16 tumors from CTR and dKO mice on day 16. Scale bar: 100 μm. n = 6. **E**, Representative images of IHC staining of HIF-1α and quantification of HIF-1α$^+$ area in subcutaneous B16 tumors from CTR and dKO mice on day 16. Scale bar: 1 mm. n = 5. Two-way ANOVA followed by Bonferroni multiple-comparison analysis for **B**. Two-tailed Student's unpaired t-test for **C, D** and **E**. Data are presented as mean ± SEM. Source data are provided as a Source Data file.

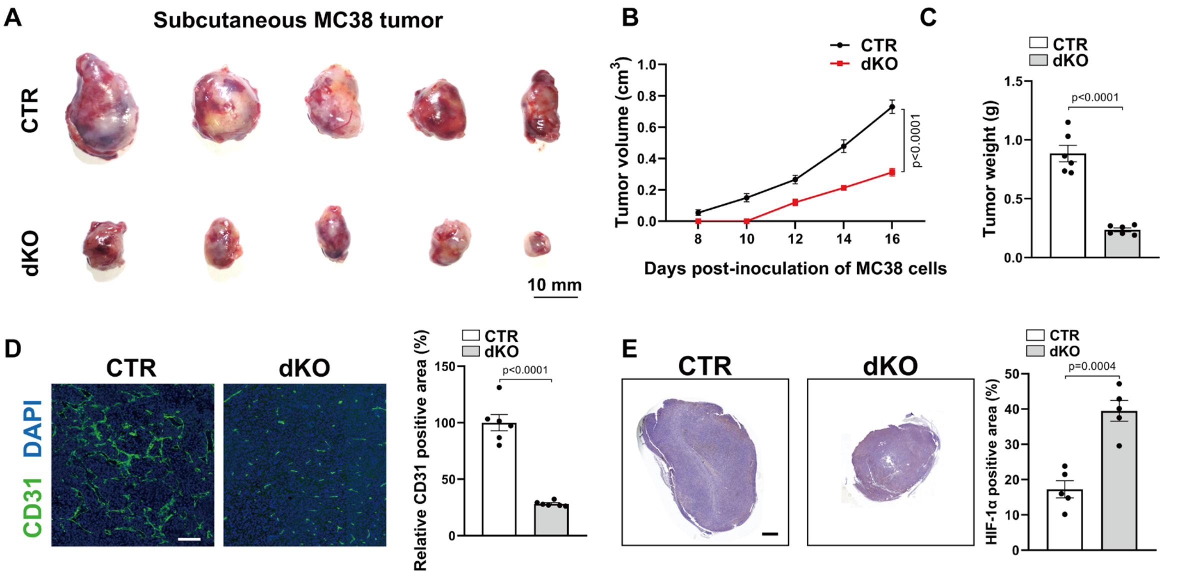

Fig. S3. Endothelial Sp1/Sp3 is necessary for MC38 tumor growth and angiogenesis. **A**, Representative images of subcutaneous MC38 tumors from CTR and dKO mice. Scale bar: 10 mm. **B**, The volume of subcutaneous MC38 tumors from CTR and dKO mice at days 8, 10, 12, 14, and 16. n = 6. **C**, The weight of subcutaneous MC-38 tumors from CTR and dKO mice on day 16. n = 6. **D**, Representative images of immunofluorescence staining of $CD31^+$ capillaries and quantification of $CD31^+$ areas in subcutaneous MC38 tumors from CTR and dKO mice on day 16. Scale bar: 100 µm. n = 6. **E**, Representative images of IHC staining of HIF-1α and quantification of HIF-1α^+ area in subcutaneous MC38 tumors from CTR and dKO mice on day 16. Scale bar: 1 mm. n = 5. Two-way ANOVA followed by Bonferroni multiple-comparison analysis for **B**. Two-tailed Student's unpaired t-test for **C, D** and **E**. Data are presented as mean ± SEM. Source data are provided as a Source Data file.

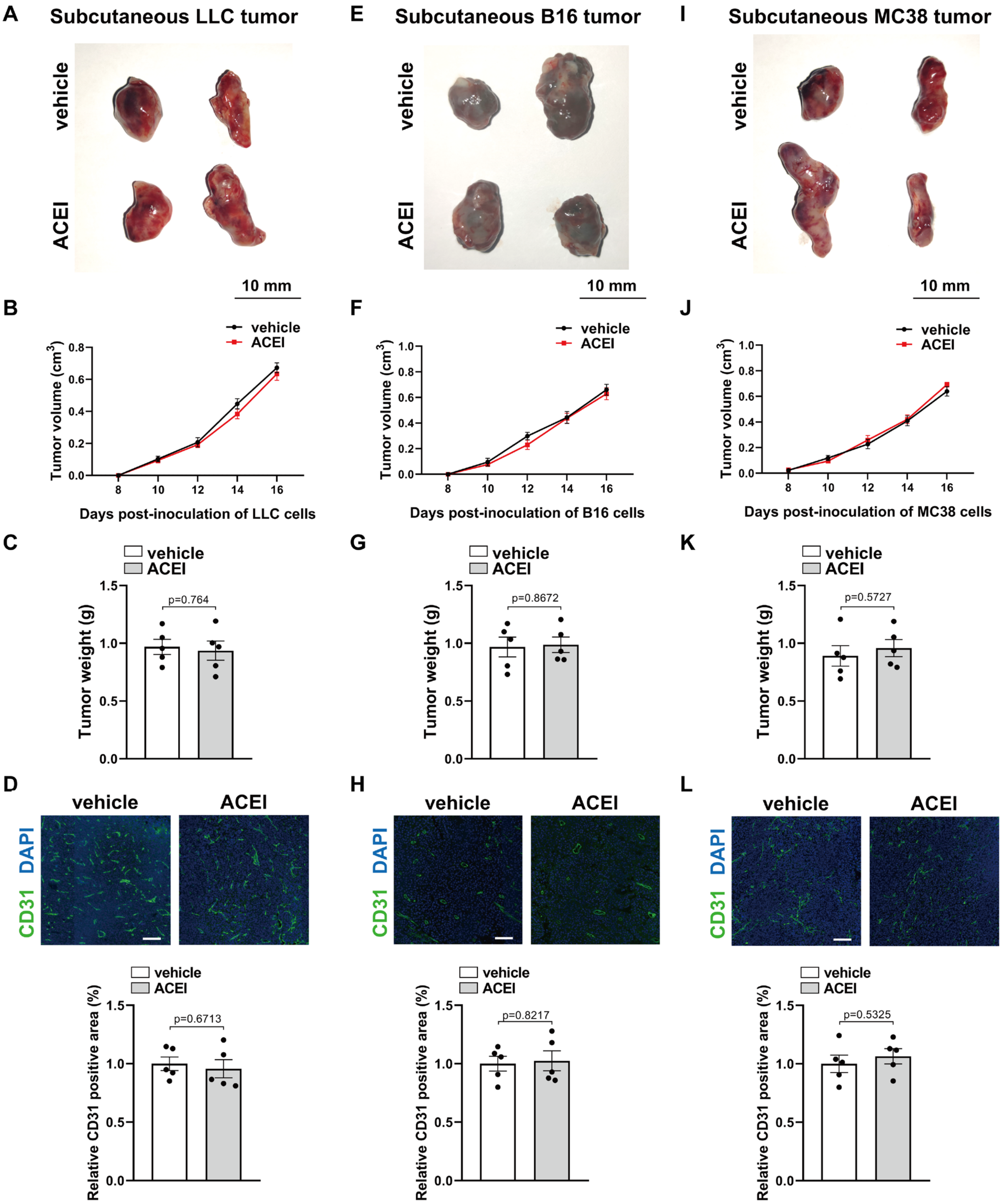
A
Subcutaneous LLC tumor
vehicle
ACEI
10 mm
B
vehicle
ACEI
Tumor volume (cm³)
Days post-inoculation of LLC cells
C
Tumor weight (g)
p=0.764
D
DAPI
CD31
Relative CD31 positive area (%)
p=0.6713
E
Subcutaneous B16 tumor
F
Days post-inoculation of B16 cells
G
p=0.8672
H
p=0.8217
I
Subcutaneous MC38 tumor
J
Days post-inoculation of MC38 cells
K
p=0.5727
L
p=0.5325

Fig. S4. ACEI has no effect on xenografted tumors growth and angiogenesis.

A, Representative images of subcutaneous LLC tumors in C57BL/6J mice treated with vehicle or ACEI. Scale bar: 10 mm. **B**, The volume of subcutaneous LLC tumors from C57BL/6J mice treated with vehicle or ACEI. measured on days 8, 10, 12, 14, and 16. n = 5. **C**, The weight of subcutaneous LLC tumors on day 16. n = 5. **D**, Representative images of immunofluorescence staining of $CD31^+$ capillaries in LLC tumors and quantification of $CD31^+$ areas. Scale bar: 100 μm. n = 5. **E**, Representative images of subcutaneous B16 tumors in C57BL/6J mice treated with vehicle or ACEI. Scale bar: 10 mm. **F**, The volume of subcutaneous B16 tumors from C57BL/6J mice treated with vehicle or ACEI. measured on days 8, 10, 12, 14, and 16. n = 5. **G**, The weight of subcutaneous B16 tumors on day 16. n = 5. **H**, Representative images of immunofluorescence staining of $CD31^+$ capillaries in B16 tumors and quantification of $CD31^+$ areas. Scale bar: 100 μm. n = 5. **I**, Representative images of subcutaneous MC38 tumors in C57BL/6J mice treated with vehicle or ACEI. Scale bar: 10 mm. **J**, The volume of subcutaneous MC38 tumors from C57BL/6J mice treated with vehicle or ACEI. measured on days 8, 10, 12, 14, and 16. n = 5. **K**, The weight of subcutaneous MC38 tumors on day 16. n = 5. **L**, Representative images of immunofluorescence staining of $CD31^+$ capillaries in MC38 tumors and quantification of $CD31^+$ areas. Scale bar: 100 μm. n = 5. Two-way ANOVA followed by Bonferroni multiple-comparison analysis for **B**, **F** and **J**. Two-tailed Student's unpaired t-test for **C, D**, **G**, **H**, **K** and **L**. Data are presented as mean ± SEM. Source data are provided as a Source Data file.

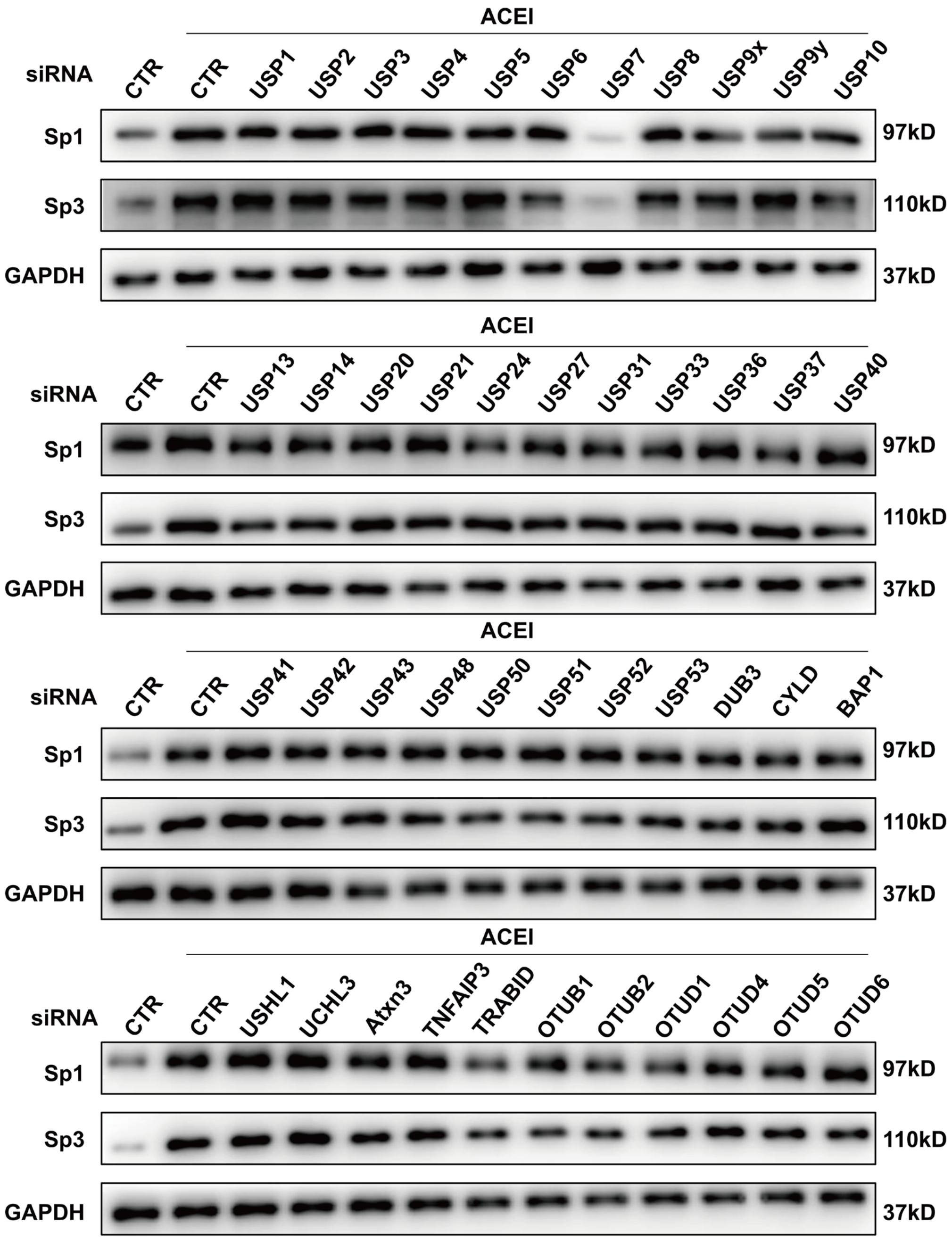

Fig. S5. USP7-deficiency by siRNA reduces the protein levels of both Sp1 and Sp3 in HUVECs.

Western blot analysis of Sp1 and Sp3 treated with siRNAs for 44 deubiquitinating enzymes in HUVECs pretreated with ACEI.

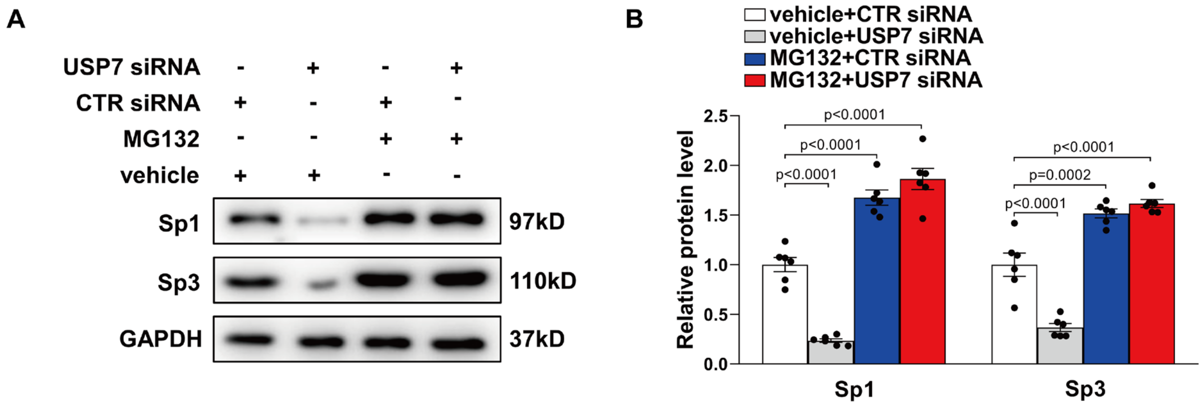

Fig. S6. MG132 assay demonstrates the ubiquitination on Sp1 and Sp3's stability. **A**, Western blot analysis of Sp1 and Sp3 protein levels in HUVECs transfected with CTR siRNA or USP7 siRNA treated with vehicle or MG132. **B**, Quantification of Sp1 or Sp3 protein level. n = 6. Two-way ANOVA followed by Bonferroni multiple comparison analysis for **B**. Data are presented as mean ± SEM. Source data are provided as a Source Data file.

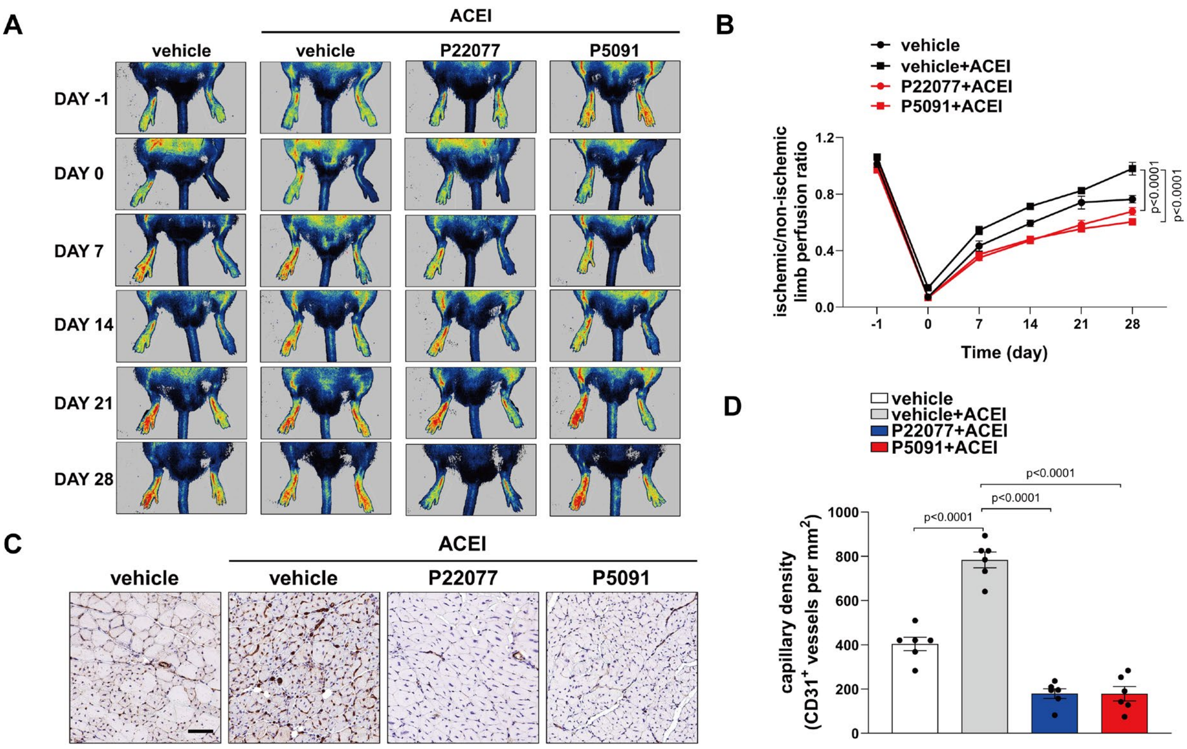

Fig. S7. ACEI increase angiogenesis after hindlimb ischemia via USP7

A, Representative laser Doppler images of the legs on day −1 (before surgery), day 0 (immediately after surgery), days 7, 14, 21, and 28 in mice treated with vehicle, ACEI + vehicle, ACEI + P22077, or ACEI + P5091. **B**, Quantification of blood flow recovery after hindlimb ischemia as determined by the ratio of foot perfusion between ischemic (left) and non-ischemic (right) legs in mice treated with vehicle, ACEI + vehicle, ACEI + P22077, or ACEI + P5091. n = 6. **C**, IHC staining analysis of $CD31^+$ staining (capillary density) in the ischemic gastrocnemius muscle. Scale bar: 50 µm. **D**, Quantification of $CD31^+$ vessels per mm^2 in **C**. n = 6. Two-way ANOVA followed by Bonferroni multiple comparison analysis for **B**. One-way ANOVA followed by Bonferroni multiple comparison analysis for **D**. Data are presented as mean ± SEM. Source data are provided as a Source Data file.

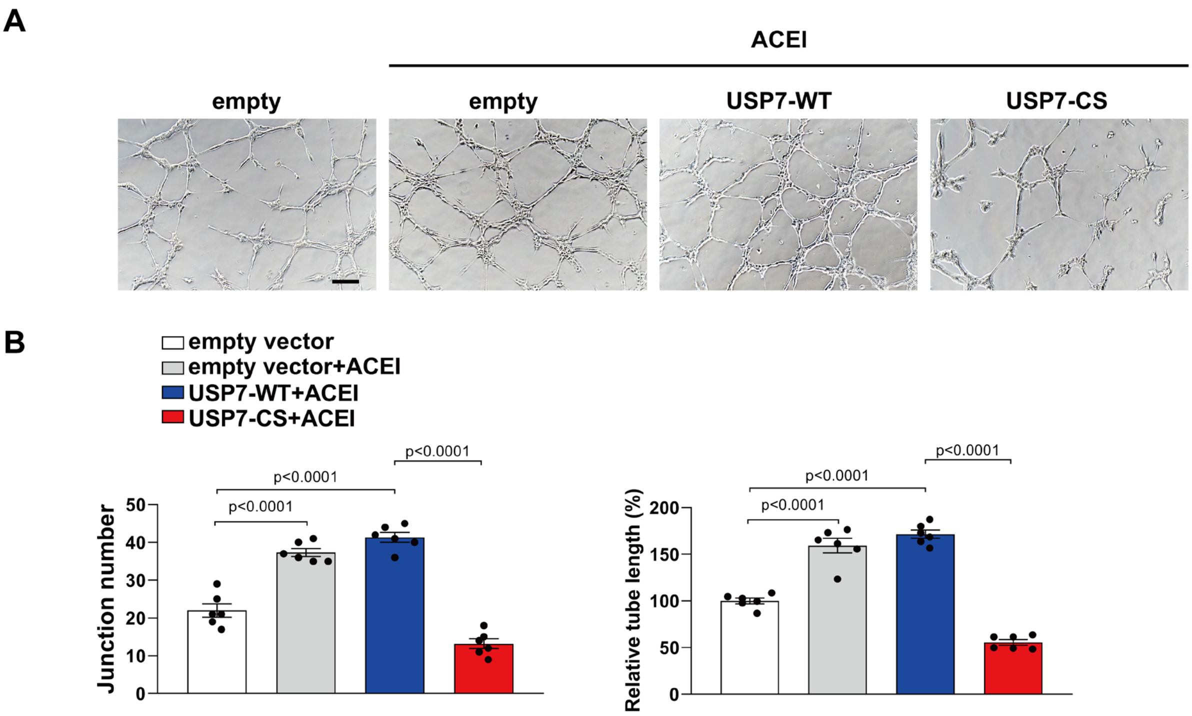

Fig. S8. Catalytic site of USP7 is necessary for ACEI-induced angiogenesis *in vitro*.

A, Capillary network formation in HUVECs transfected with empty vector, USP7-WT, or USP7-CS (C233S) in combination with or without ACEI on Matrigel. Scale bar: 50 μm. **B**, Quantification of the junction number and relative tube length in HUVECs of **A**. n = 6. Two-way ANOVA followed by Bonferroni multiple comparison analysis for **B**. Data are presented as mean ± SEM. Source data are provided as a Source Data file.

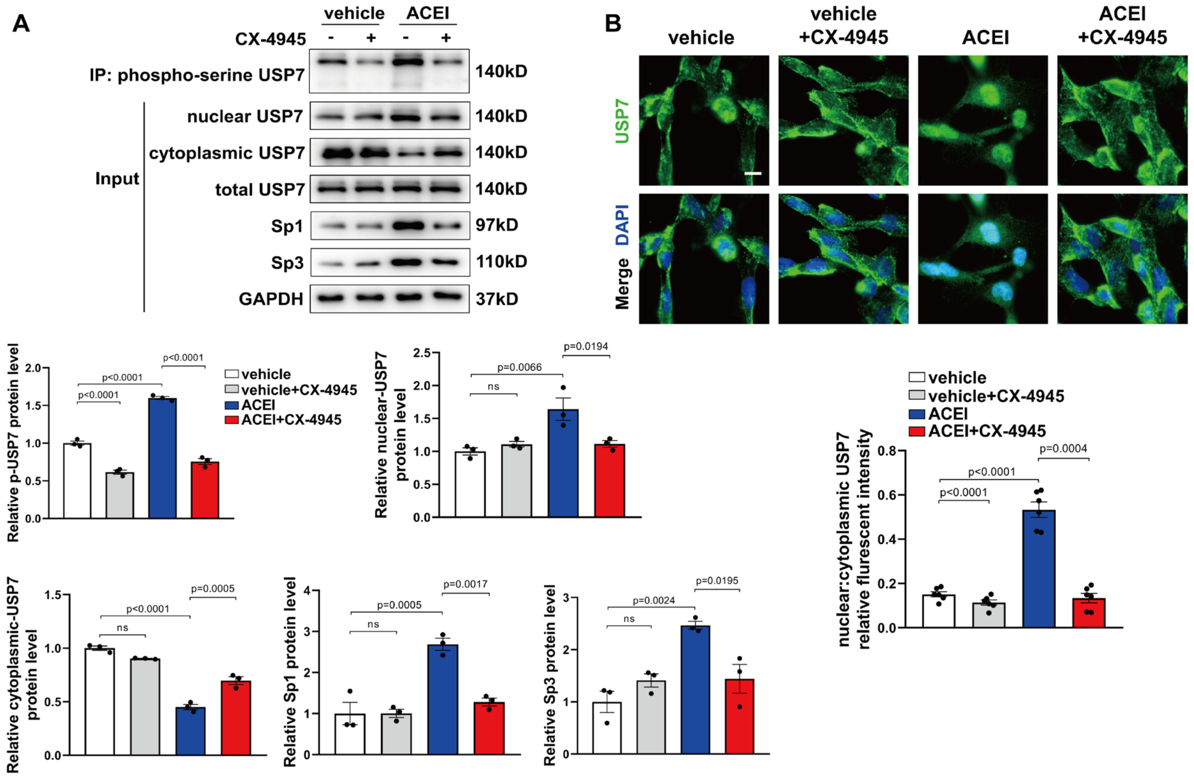

Fig. S9. ACEI regulate USP7 via CK2-mediated phosphorylation

A, Representative western blot analysis of USP7 after anti-phospho-serine immunoprecipitation of HUVECs treated with vehicle or ACEI, as indicated in combination with or without CX-4945 (inhibitor against catalytic CK2α and CK2α’ subunits). The protein levels of USP7 were measured by western blot analysis in nuclear, cytoplasmic, and whole-cell lysates (total USP7). The protein level of Sp1 and Sp3 were measured by western blot analysis. Bottom, quantification of the western analysis. n = 3. **B**, Representative confocal microscopy images of immunofluorescence staining for USP7 and DAPI in HUVECs treated with vehicle or ACEI, as indicated in combination with or without CX-4945. Scale bar: 20 μm. Bottom, quantification of the ratio of USP7 in the nuclear to cytoplasmic fractions. n = 6.

One-way ANOVA followed by Bonferroni multiple comparison analysis for **A** and **B**. Data are presented as mean ± SEM. Source data are provided as a Source Data file.

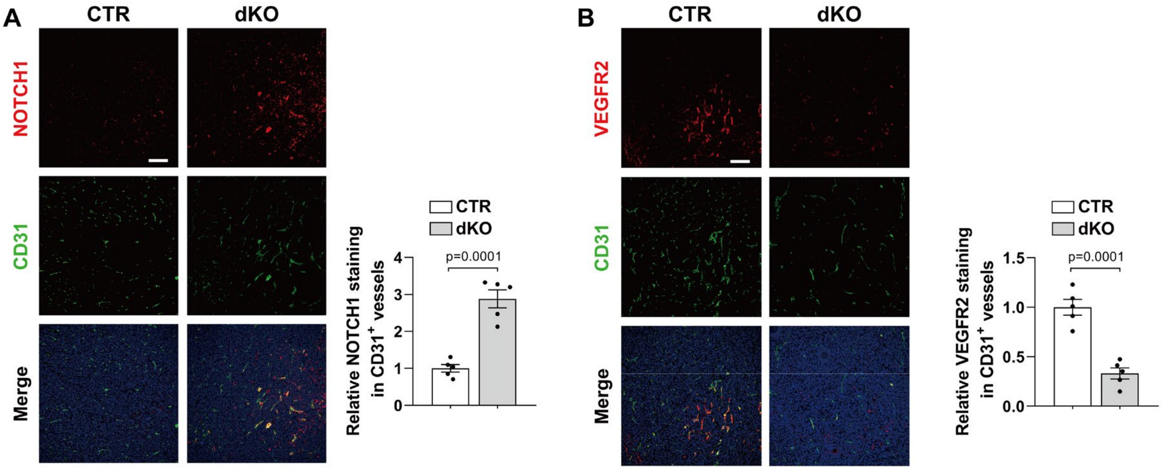

Fig. S10. Immunofluorescence staining for Notch1 and VEGFR2 in LLC xenograft tumors

A, Representative microscopy images and quantification of immunofluorescence staining for Notch1 and CD31 in LLC xenograft tumors from CTR and dKO mice. Scale bar: 30 μm. **B**, Representative microscopy images and quantification of immunofluorescence staining for VEGFR2 and CD31 in LLC xenograft tumors from CTR and dKO mice. Scale bar: 30 μm. n = 5. Two-tailed Student's unpaired t-test for **A** and **B**. Data are presented as mean ± SEM. Source data are provided as a Source Data file.

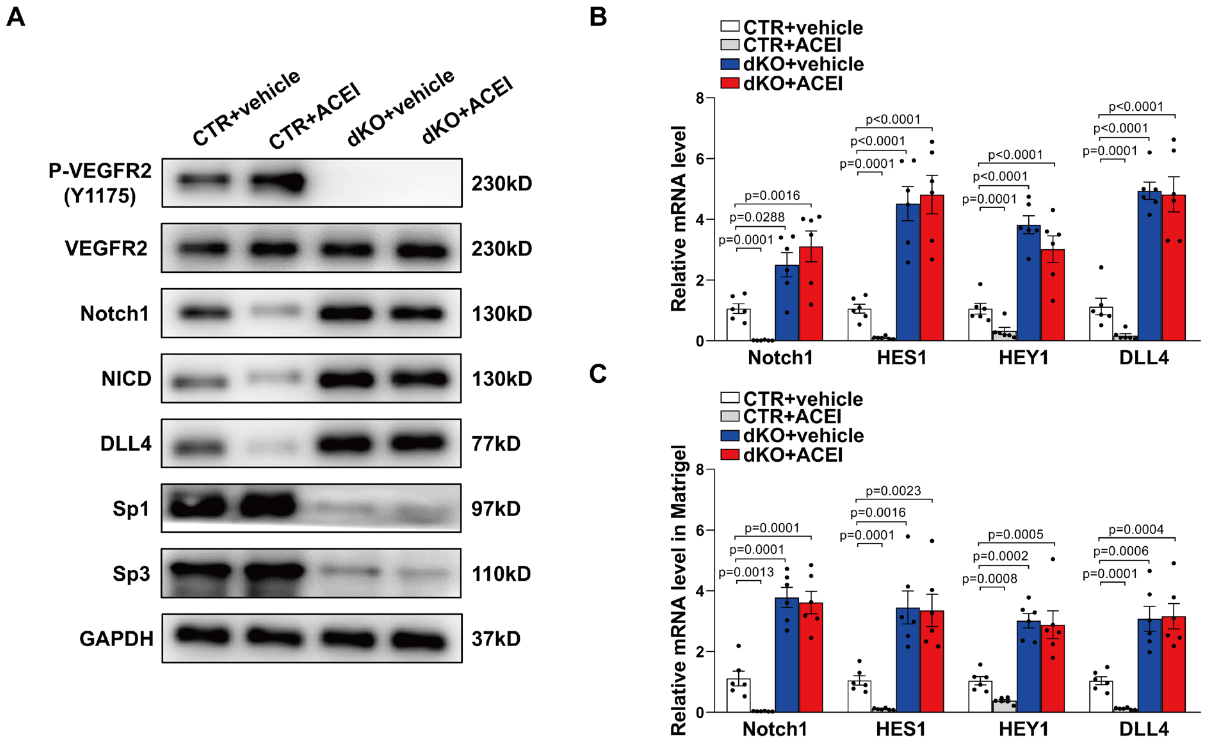

Fig. S11. ACEI regulate Sp1/Sp3-Notch1-VEGFR2 signaling

A, Western blot analysis of P-VEGFR2 (Y1175), VEGFR2, Notch1, NICD, DLL4, Sp1, and Sp3 protein levels in isolated retinal endothelial cells from CTR and dKO mice treated with vehicle or ACEI. **B**, qPCR analysis of Notch1, HES1, HEY1, and DLL4 mRNA levels in isolated retinal endothelial cells from CTR and dKO mice treated with vehicle or ACEI. **C**, mRNA expression levels of Notch1, HES1, HEY1, and DLL4 assessed using qPCR analysis in the Matrigel plugs retrieved from CTR or dKO mice treated with or without ACEI. n = 6. Two-way ANOVA followed by Bonferroni multiple comparison analysis was performed for **B** and **C**. Data are presented as mean ± SEM. Source data are provided as a Source Data file.

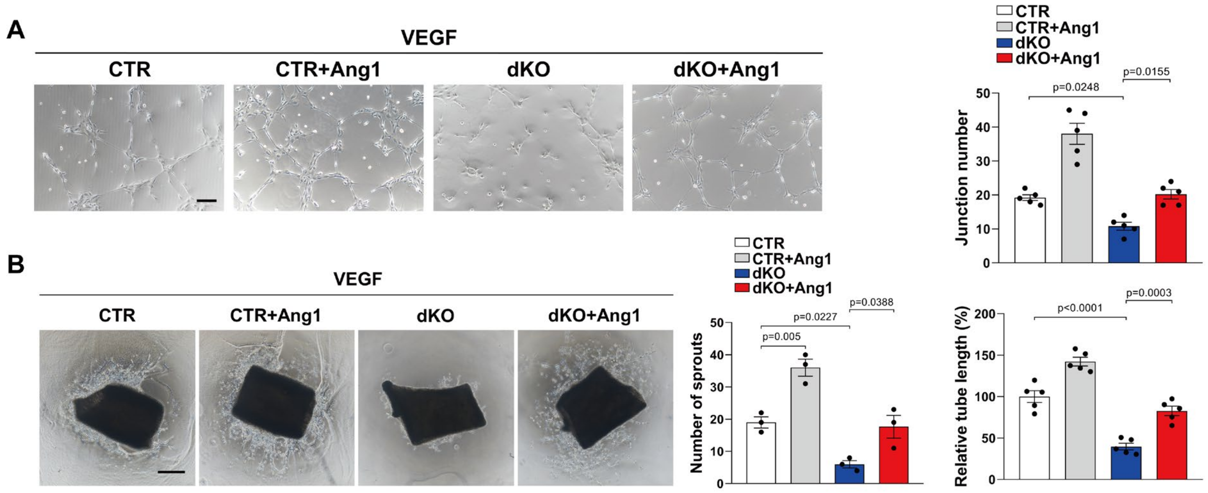

Fig. S12. Combined action of VEGF and Ang1 promote angiogenesis in dKO MLECs

A, Capillary network formation in primary MLECs (mouse lung endothelial cells) from CTR and dKO mice treated with VEGF in combination with or without Ang1 (angiopoietin 1) on Matrigel. Right, quantification of the junction number and relative tube length. n = 5. Scale bar: 50 μm. **B**. Aortic ring assays showing the number of aortic sprouts from CTR and dKO mice treated with VEGF in combination with or without Ang1 embedded in Matrigel for 6 days. Right, quantification of sprout number. n = 3. Scale bar: 500 μm. One-way ANOVA followed by Bonferroni multiple comparison analysis for **A** and **B**. Data are presented as mean ± SEM. Source data are provided as a Source Data file.

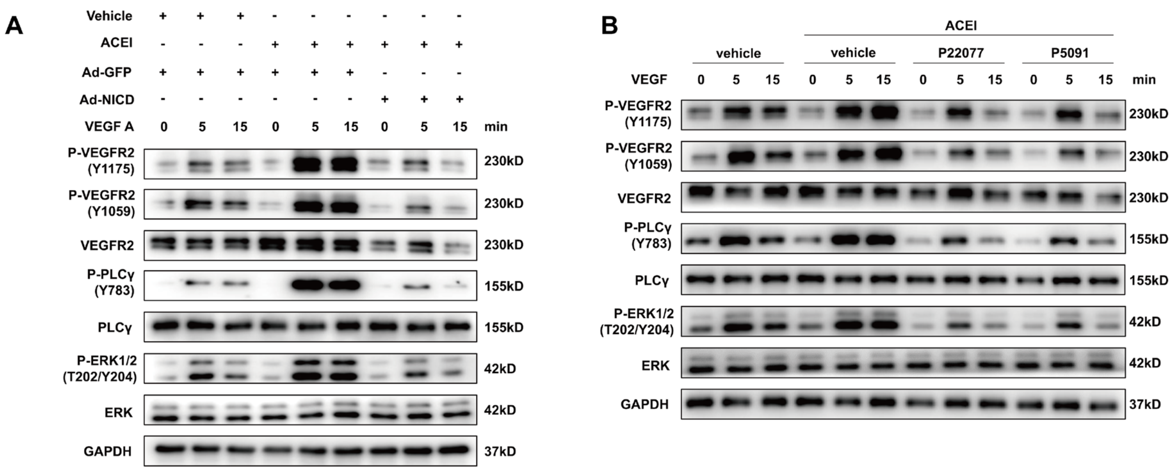

Fig. S13. ACEI activates VEGFR2 via USP7-Sp1/Sp3-Notch1 signaling

A, Western blot analysis of phosphorylated VEGFR2 (Y1175, Y1059), VEGFR2, PLCγ (Y783), PLCγ, ERK1/2 (Thr202/Tyr204) and ERK1/2 in Ad-GFP or Ad-NICD with different treatments. **B**, Western blot analysis of phosphorylated VEGFR2 (Y1175, Y1059), VEGFR2, PLCγ (Y783), PLCγ, ERK1/2 (Thr202/Tyr204) and ERK1/2 in Ad-GFP or Ad-NICD with different treatments.

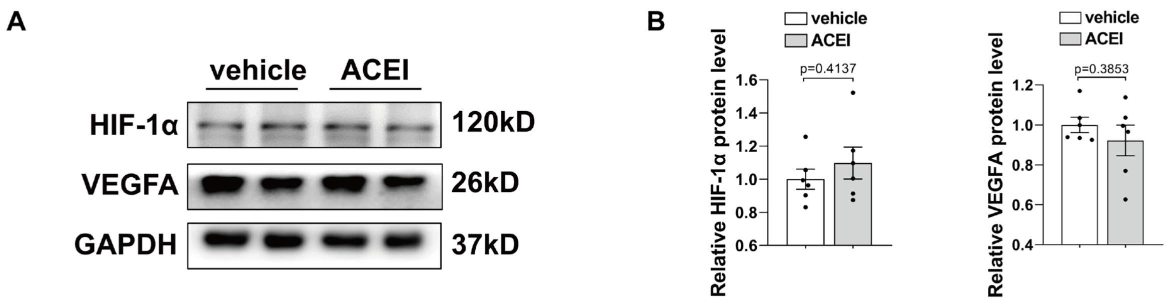

Fig. S14. ACEI have no effects on the protein levels of HIF1-α and VEGFA in ECs.

A, Western blot of HIF1-α and VEGFA in HUVECs treated with vehicle or ACEI. **B**, Quantification of western blot in HUVECs of **A**. n = 6. Two-tailed Student's unpaired t-test for **B**. Data are presented as mean ± SEM. Source data are provided as a Source Data file.

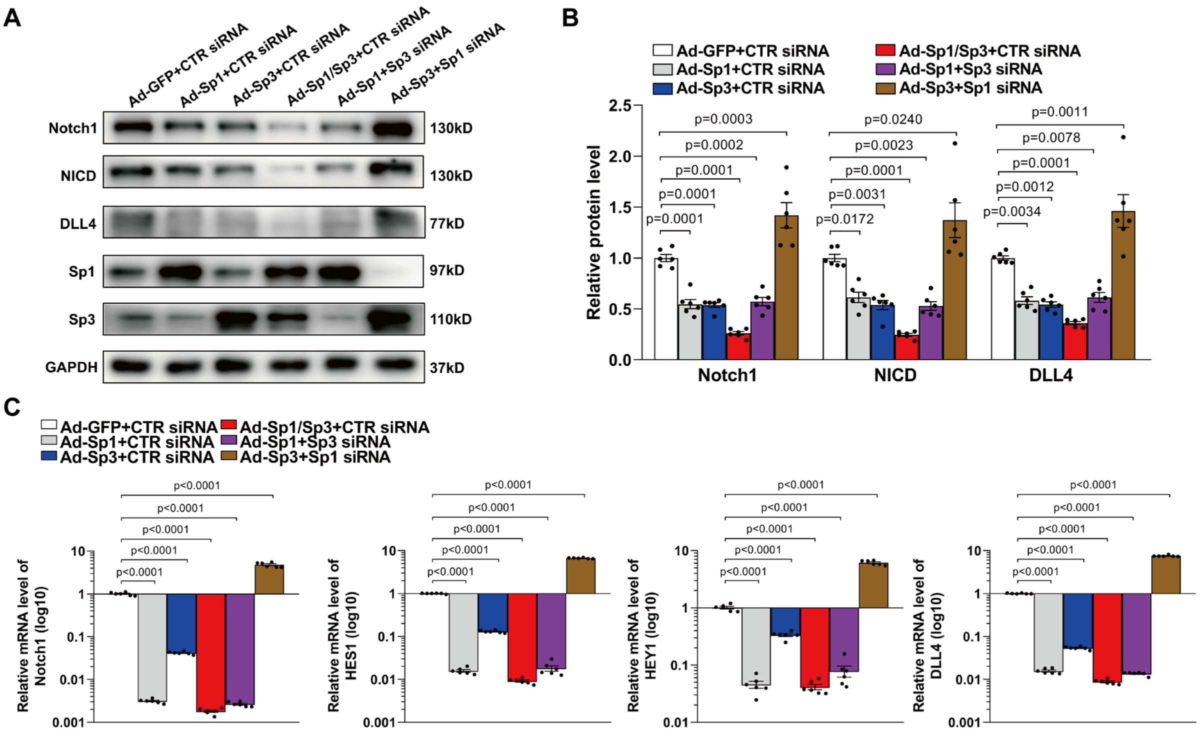

Fig. S15. Sp3 enhances Sp1-mediated repression activity of Notch1 in ECs

A, Western blot of Notch1, NICD, DLL4, Sp1, and Sp3 protein levels in HUVECs transfected with Ad-GFP + CTR siRNA, Ad-Sp1 + CTR siRNA, Ad-Sp3 + CTR siRNA, Ad-Sp1/Sp3 + CTR siRNA, Ad-Sp1 + Sp3 siRNA, or Ad-Sp3 + Sp1 siRNA. **B**, Quantification of western blot in HUVECs of **A**. **C**, qPCR analysis of Notch1, HES1, HEY1, and DLL4 mRNA levels in HUVECs transfected with Ad-GFP + CTR siRNA, Ad-Sp1 + CTR siRNA, Ad-Sp3 + CTR siRNA, Ad-Sp1/Sp3 + CTR siRNA, Ad-Sp1 + Sp3 siRNA, or Ad-Sp3 + Sp1 siRNA. n = 6. One-way ANOVA followed by Bonferroni multiple-comparison analysis was used for **B** and **C**. Data are presented as mean ± SEM. Source data are provided as a Source Data file.

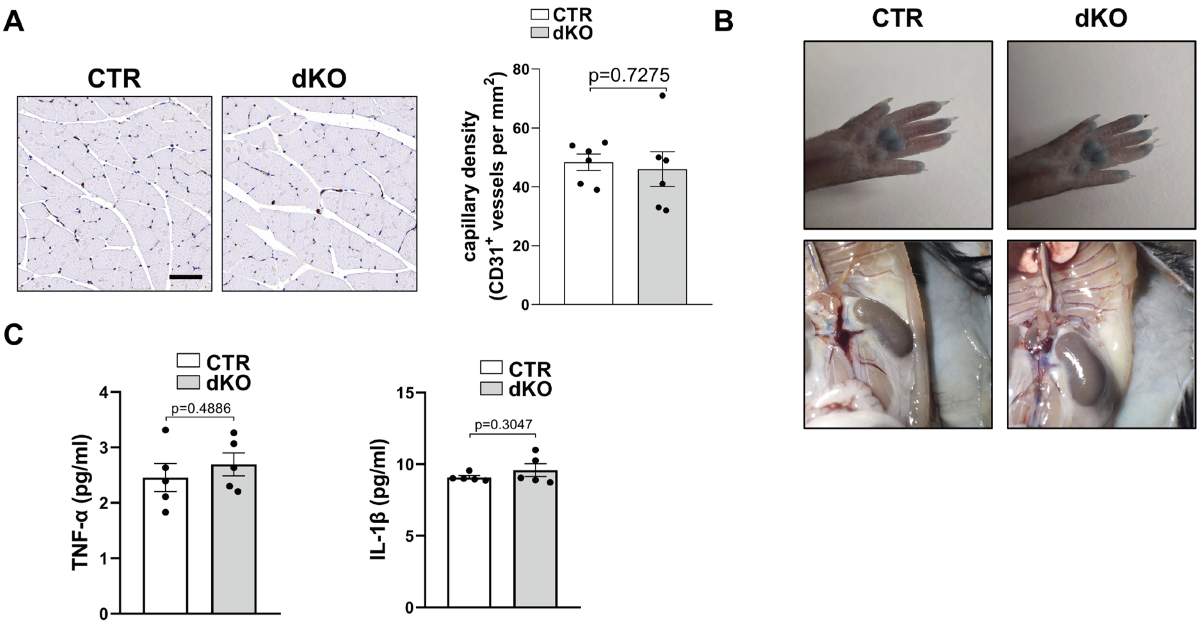

Fig. S16. Basal vessel density, vascular permeability and inflammation level in mice

A, IHC analysis of $CD31^+$ staining (capillary density) in the gastrocnemius muscle before ligating the femoral artery. Right, quantification of $CD31^+$ vessels per mm^2 in CTR and dKO mice. n=6. Scale bar: 50 μm. **B**, Microvascular permeability measured by Evans blue content in CTR and dKO mice. **C**, Serum IL-1β and TNF-α in CTR and dKO mice. n=5. Two-tailed Student's unpaired t-test for **A** and **C**. Data are presented as mean ± SEM. Source data are provided as a Source Data file.
