# Supplementary material for: Angiotensin-converting enzyme inhibitor promotes angiogenesis through Sp1/Sp3-mediated inhibition of notch signaling in male mice
Source: Nat Commun. 2023 Feb 9;14:731. doi: 10.1038/s41467-023-36409-z (PMC9911748; doi:10.1038/s41467-023-36409-z)
Supplement: Supplementary file 3 — Reporting Summary [file 41467_2023_36409_MOESM3_ESM.pdf]

nature portfolio

Corresponding author(s): Wencheng Zhang

Last updated by author(s): Jan 20, 2023

Reporting Summary

Nature Portfolio wishes to improve the reproducibility of the work that we publish. This form provides structure for consistency and transparency in reporting. For further information on Nature Portfolio policies, see our Editorial Policies and the Editorial Policy Checklist.

Statistics

For all statistical analyses, confirm that the following items are present in the figure legend, table legend, main text, or Methods section.

n/a	Confirmed	
☐	☒	The exact sample size (*n*) for each experimental group/condition, given as a discrete number and unit of measurement
☐	☒	A statement on whether measurements were taken from distinct samples or whether the same sample was measured repeatedly
☐	☒	The statistical test(s) used AND whether they are one- or two-sided<br>*Only common tests should be described solely by name; describe more complex techniques in the Methods section.*
☒	☐	A description of all covariates tested
☐	☒	A description of any assumptions or corrections, such as tests of normality and adjustment for multiple comparisons
☐	☒	A full description of the statistical parameters including central tendency (e.g. means) or other basic estimates (e.g. regression coefficient) AND variation (e.g. standard deviation) or associated estimates of uncertainty (e.g. confidence intervals)
☐	☒	For null hypothesis testing, the test statistic (e.g. *F*, *t*, *r*) with confidence intervals, effect sizes, degrees of freedom and *P* value noted<br>*Give P values as exact values whenever suitable.*
☒	☐	For Bayesian analysis, information on the choice of priors and Markov chain Monte Carlo settings
☒	☐	For hierarchical and complex designs, identification of the appropriate level for tests and full reporting of outcomes
☒	☐	Estimates of effect sizes (e.g. Cohen's *d*, Pearson's *r*), indicating how they were calculated

Our web collection on statistics for biologists contains articles on many of the points above.

Software and code

Policy information about availability of computer code

Data collection	The softwares to collect the data: Amersham Imager 680; LightCycler 480 II;Pannoramic Scan; Leica H-700FA; PeriCam PSI Z, Perimed;
Data analysis	The softwares to analyse the data: SPSS23.0; Graphpad Prism 8; Image J v18.0; Image Pro Plus 6.0.

For manuscripts utilizing custom algorithms or software that are central to the research but not yet described in published literature, software must be made available to editors and reviewers. We strongly encourage code deposition in a community repository (e.g. GitHub). See the Nature Portfolio guidelines for submitting code & software for further information.

Data

Policy information about availability of data

All manuscripts must include a data availability statement. This statement should provide the following information, where applicable:

- Accession codes, unique identifiers, or web links for publicly available datasets
- A description of any restrictions on data availability
- For clinical datasets or third party data, please ensure that the statement adheres to our policy

Data supporting the findings of this study are available within the article and its Supplementary Information files. Source data are provided with this paper.

Human research participants

Policy information about studies involving human research participants and Sex and Gender in Research.

Reporting on sex and gender	The sex was not condered in the analysis.
Population characteristics	Human tissue biopsies from gastrocnemius were obtained from the Qilu Hospital of Shandong University during surgery. 5 patients with CLI with an average of 56 were included in this study. 5 patients without CLI with an average of 59 were included in this study.
Recruitment	These patients with CLI were recruited while in hospital undergoing above or below knee amputation. The patients without CLI were recruited from the Qilu Hospital of Shandong University during surgery. The sex and age was not considered in the analysis.
Ethics oversight	All experimental protocols were approved by the Ethics Committee of Qilu Hospital of Shandong University.

Note that full information on the approval of the study protocol must also be provided in the manuscript.

Field-specific reporting

Please select the one below that is the best fit for your research. If you are not sure, read the appropriate sections before making your selection.

☒ Life sciences ☐ Behavioural & social sciences ☐ Ecological, evolutionary & environmental sciences

For a reference copy of the document with all sections, see nature.com/documents/nr-reporting-summary-flat.pdf

Life sciences study design

All studies must disclose on these points even when the disclosure is negative.

Sample size	We used standard sample sizes reported in the literature previously in mouse studies. The sample size of animal experiments according to similar studies in the field was chose and at least 5 mice per group was used. The number of the independent experiments for cell and biological experiments was indicated in each figure legend.
Data exclusions	Except where experiments failed because of technical issues, no data was excluded.
Replication	All in vivo and in vitro experiments were highly reproducible and were independently repeated at least 3 times. All experiments were performed independently multiple times using biologically independent replicates. All attempts at replication were successful.
Randomization	Mice were randomly assigned to groups. Cells were grown under the same conditions and randomly allocated into different groups without any bias.
Blinding	The investigators were blinded to group allocation during data collection and analysis. We collected and analyzed the compared samples under the same conditions.

Reporting for specific materials, systems and methods

We require information from authors about some types of materials, experimental systems and methods used in many studies. Here, indicate whether each material, system or method listed is relevant to your study. If you are not sure if a list item applies to your research, read the appropriate section before selecting a response.

Materials & experimental systems

n/a	Involved in the study	
☐	☒	Antibodies
☐	☒	Eukaryotic cell lines
☒	☐	Palaeontology and archaeology
☐	☒	Animals and other organisms
☒	☐	Clinical data
☒	☐	Dual use research of concern

Methods

n/a	Involved in the study	
☒	☐	ChIP-seq
☒	☐	Flow cytometry
☒	☐	MRI-based neuroimaging

Antibodies

Antibodies used	1. GAPDH, RRID: AB_10622025, Cell Signaling Technology, Catalog number: 5174, Clone ID: D16H11, Host organism: rabbit. Target antigen: human, mouse, rat, monkey. Applications: W, IHC-P, IF-IC.<br>2. Notch1, RRID: AB_2153354, Cell Signaling Technology, Catalog number: 3608. Host organism: rabbit. Target antigen: human, mouse, rat. Applications: W, IP, IHC-P, ChIP.<br>3. DLL4, RRID: AB_2800263, Cell Signaling Technology, Catalog number: 96406, Clone ID: D7N3H. Host organism: rabbit. Target antigen: human. Applications: W, IP, IF-IC.<br>4. Cleaved Notch1, RRID: AB_2153348, Cell Signaling Technology, Catalog number: 4147, Clone ID: D3B8. Host organism: rabbit. Target antigen: human, mouse, rat. Applications: W, IP, ChIP.<br>5. Phospho-VEGF Receptor 2 (Tyr1175), RRID: AB_331377, Cell Signaling Technology, Catalog number: 2478, Clone ID: 19A10. Host organism: rabbit. Target antigen: human, mouse. Applications: W, IHC-P, IF-IC.<br>6. VEGF Receptor 2, RRID: AB_2212507, Cell Signaling Technology, Catalog number: 2479, Clone ID:55B11. Host organism: rabbit. Target antigen: human, mouse. Applications: W, IP, IHC-P, IF-F, IF-IC.<br>7. Phospho-p44/42 MAPK (Erk1/2) (Thr202/Tyr204), RRID: AB_2315112, Cell Signaling Technology, Catalog number: 4370, Clone ID: D13.14.4E. Host organism: rabbit. Target antigen: human, mouse, rat, hamster, monkey, mink, d. melanogaster, zebrafish, bovine, dog, pig, s. cerevisiae. Applications: W, IP, IHC-P, IF-IC, F.<br>8. Phospho-PLCγ1 (Tyr783), RRID: AB_2728690, Cell Signaling Technology, Catalog number: 14008, Clone ID: D6M9S. Host organism: rabbit. Target antigen: human, mouse. Applications: W, IP, F.<br>9. PLCγ1, RRID: AB_10691383, Cell Signaling Technology, Catalog number: 5690, Clone ID: D9H10. Host organism: rabbit. Target antigen: human, mouse, rat, monkey. Applications: W, IP, IHC-P.<br>10. Ubiquitin, RRID: AB_11181462, Cell Signaling Technology, Catalog number: 3936, Clone ID: P4D1. Host organism: mouse. Target antigen: All. Applications: W, IHC-P.<br>11. DYKDDDDK Tag, RRID: AB_2572291, Cell Signaling Technology, Catalog number: 14793, Clone ID: D6W5B. Host organism: rabbit. Target antigen: All. Applications: W, IP, IHC-P, IF-IC, F, ChIP.<br>12. HA-Tag, RRID: AB_1549585, Cell Signaling Technology, Catalog number: 3724, Clone ID: C29F4. Host organism: rabbit. Target antigen: All. Applications: W, IP, IHC-P, IF-IC, F, ChIP.<br>13. Myc-Tag, RRID: AB_1549585, Cell Signaling Technology, Catalog number: 3724, Clone ID: C29F4. Host organism: mouse. Target antigen: All. Applications: W, IP, IHC-P, IF-IC, F, ChIP.<br>14. USP7, RRID: AB_203276, Bethyl, Catalog number: A300-033A-T. Host organism: rabbit. Target antigen: human, mouse. Applications: W, IP, IHC.<br>15. Sp1, RRID: AB_310773, Millipore, Catalog number: 07-645. Host organism: rabbit. Target antigen: human, mouse, rat. Applications: W, IP, IHC-P, IF-IC, F, ChIP.<br>16. Sp3, Abcam, Catalog number: ab227856. Host organism: rabbit. Target antigen: human. Applications: WB, IP, IHC-P, ICC/IF.<br>17. CD31, RRID: AB_726362, Abcam, Catalog number: ab28364. Host organism: rabbit. Target antigen: mouse, human, pig. Applications: IHC-Fr, IHC-P, ICC/IF, IHC-FoFr, WB.<br>18. VEGFA, RRID: AB_2212642, Abcam, Catalog number: ab46154. Host organism: rabbit. Target antigen: mouse, human. Applications: WB, ELISA.<br>19. HIF-1α, RRID: AB_2622225, Cell Signaling Technology, Catalog number: 14179, Clone ID: D2U3T. Host organism: rabbit. Target antigen: human, mouse, rat, monkey. Applications: WB, ChIP.<br>20. phosphoserine/threonine, RRID: AB_1184778, ECM Biosciences, Catalog number: PP2551, Host organism: rabbit. Target antigen: human. Applications: WB, IP, ELISA.<br>21. Normal Rabbit IgG, RRID: AB_1031062, Cell Signaling Technology, Catalog number: 2729, Host organism: rabbit.<br>22. HRP-conjugated Affinipure Goat Anti-Mouse IgG(H+L), RRID: AB_2722565, Proteintech, Catalog number: SA00001-1, dilution: 1:5000.<br>23. HRP-conjugated Affinipure Goat Anti-Rabbit IgG(H+L), Proteintech, RRID: AB_2722564, Catalog number: SA00001-2, dilution: 1:5000.
Validation	1. GAPDH, RRID: AB_10622025, Cell Signaling Technology, Catalog number: 5174, Clone ID: D16H11, Host organism: rabbit. Target antigen: human, mouse, rat, monkey. Applications: W, IHC-P, IF-IC.<br>2. Notch1, RRID: AB_2153354, Cell Signaling Technology, Catalog number: 3608. Host organism: rabbit. Target antigen: human, mouse, rat. Applications: W, IP, IHC-P, ChIP.<br>3. DLL4, RRID: AB_2800263, Cell Signaling Technology, Catalog number: 96406, Clone ID: D7N3H. Host organism: rabbit. Target antigen: human. Applications: W, IP, IF-IC.<br>4. Cleaved Notch1, RRID: AB_2153348, Cell Signaling Technology, Catalog number: 4147, Clone ID: D3B8. Host organism: rabbit. Target antigen: human, mouse, rat. Applications: W, IP, ChIP.<br>5. Phospho-VEGF Receptor 2 (Tyr1175), RRID: AB_331377, Cell Signaling Technology, Catalog number: 2478, Clone ID: 19A10. Host organism: rabbit. Target antigen: human, mouse. Applications: W, IHC-P, IF-IC.<br>6. VEGF Receptor 2, RRID: AB_2212507, Cell Signaling Technology, Catalog number: 2479, Clone ID:55B11. Host organism: rabbit. Target antigen: human, mouse. Applications: W, IP, IHC-P, IF-F, IF-IC.<br>7. Phospho-p44/42 MAPK (Erk1/2) (Thr202/Tyr204), RRID: AB_2315112, Cell Signaling Technology, Catalog number: 4370, Clone ID: D13.14.4E. Host organism: rabbit. Target antigen: human, mouse, rat, hamster, monkey, mink, d. melanogaster, zebrafish, bovine, dog, pig, s. cerevisiae. Applications: W, IP, IHC-P, IF-IC, F.<br>8. Phospho-PLCγ1 (Tyr783), RRID: AB_2728690, Cell Signaling Technology, Catalog number: 14008, Clone ID: D6M9S. Host organism: rabbit. Target antigen: human, mouse. Applications: W, IP, F.<br>9. PLCγ1, RRID: AB_10691383, Cell Signaling Technology, Catalog number: 5690, Clone ID: D9H10. Host organism: rabbit. Target antigen: human, mouse, rat, monkey. Applications: W, IP, IHC-P.<br>10. Ubiquitin, RRID: AB_11181462, Cell Signaling Technology, Catalog number: 3936, Clone ID: P4D1. Host organism: mouse. Target antigen: All. Applications: W, IHC-P.<br>11. DYKDDDDK Tag, RRID: AB_2572291, Cell Signaling Technology, Catalog number: 14793, Clone ID: D6W5B. Host organism: rabbit. Target antigen: All. Applications: W, IP, IHC-P, IF-IC, F, ChIP.

12. HA-Tag, RRID: AB_1549585, Cell Signaling Technology, Catalog number: 3724, Clone ID: C29F4. Host organism: rabbit. Target antigen: All. Applications: W, IP, IHC-P, IF-IC, F, ChIP.
13. Myc-Tag, RRID: AB_1549585, Cell Signaling Technology, Catalog number: 3724, Clone ID: C29F4. Host organism: mouse. Target antigen: All. Applications: W, IP, IHC-P, IF-IC, F, ChIP.
14. USP7, RRID: AB_203276, Bethyl, Catalog number: A300-033A-T. Host organism: rabbit. Target antigen: human, mouse. Applications: W, IP, IHC.
15. Sp1, RRID: AB_310773, Millipore, Catalog number: 07-645. Host organism: rabbit. Target antigen: human, mouse, rat. Applications: W, IP, IHC-P, IF-IC, F, ChIP.
16. Sp3, Abcam, Catalog number: ab227856. Host organism: rabbit. Target antigen: human. Applications: WB, IP, IHC-P, ICC/IF.
17. CD31, RRID: AB_726362, Abcam, Catalog number: ab28364. Host organism: rabbit. Target antigen: mouse, human, pig. Applications: IHC-Fr, IHC-P, ICC/IF, IHC-FoFr, WB.
18. VEGFA, RRID: AB_2212642, Abcam, Catalog number: ab46154. Host organism: rabbit. Target antigen: mouse, human. Applications: WB, ELISA.
19. HIF-1α, RRID: AB_2622225, Cell Signaling Technology, Catalog number: 14179, Clone ID: D2U3T. Host organism: rabbit. Target antigen: human, mouse, rat, monkey. Applications: WB, ChIP.
20. phosphoserine/threonine, RRID: AB_1184778, ECM Biosciences, Catalog number: PP2551, Host organism: rabbit. Target antigen: human. Applications: WB, IP, ELISA.
21. Normal Rabbit IgG, RRID: AB_1031062, Cell Signaling Technology, Catalog number: 2729, Host organism: rabbit.
22. HRP-conjugated Affinipure Goat Anti-Mouse IgG(H+L), RRID: AB_2722565, Proteintech, Catalog number: SA00001-1, dilution: 1:5000.
23. HRP-conjugated Affinipure Goat Anti-Rabbit IgG(H+L), Proteintech, RRID: AB_2722564, Catalog number: SA00001-2, dilution: 1:5000.

Eukaryotic cell lines

Policy information about cell lines and Sex and Gender in Research

Cell line source(s)	The HEK 293T/17 cell line (CRL-11268), LLC cell line, B16 cell line and MC38 cell line were purchased from ATCC. HUVEC cells were isolated from normal female human umbilical veins, which were collected from Qilu Hospital of Shandong University. Mouse lung endothelial cells and mouse retinal endothelial cells were isolated from indicated mouse.
Authentication	The cell lines were not authenticated.
Mycoplasma contamination	All cell lines tested negative for mycoplasma contamination.
Commonly misidentified lines (See ICLAC register)	No commonly misidentified cell lines are used in the study.

Animals and other research organisms

Policy information about studies involving animals; ARRIVE guidelines recommended for reporting animal research, and Sex and Gender in Research

Laboratory animals	The animals we used were all male mice of eight weeks and P5. The strains included VE-CAD-CreERT2, VE-CAD-CreERT2+/Sp1(flox/flox)/Sp3(flox/flox), VE-CAD-CreERT2-/Sp1(flox/flox)/Sp3(flox/flox), VE-CAD-CreERT2+/Sp1(flox/flox), VE-CAD-CreERT2+/Sp3(flox/flox) and C57BL/6J. Mice were housed on a 12-h light/dark cycle and given ad libitum access to food and water. All animal protocols were approved by the Institutional Animal Care and Use Committee of Cheeloo College of Medicine, Shandong University. Animal vivarium condition; light/dark cycle: 12 hours, temperature: 18-23 degrees C, humidity:40-60 %
Wild animals	The study did not involve wild animals.
Reporting on sex	The animals we used were all male mice according to similar studies in the field, so the findings in vivo applied to only male mice.
Field-collected samples	The study did not involve samples collected from the field.
Ethics oversight	Institutional Animal Care and Use Committee of Cheeloo College of Medicine, Shandong University.

Note that full information on the approval of the study protocol must also be provided in the manuscript.
